# Flame Image Processing and Classification Using a Pre-Trained VGG16 Model in Combustion Diagnosis

**DOI:** 10.3390/s21020500

**Published:** 2021-01-12

**Authors:** Zbigniew Omiotek, Andrzej Kotyra

**Affiliations:** Faculty of Electrical Engineering and Computer Science, Lublin University of Technology, 20-618 Lublin, Poland; a.kotyra@pollub.pl

**Keywords:** image processing, flame segmentation, classification, deep learning, VGG16, convolutional neural networks, industrial combustion

## Abstract

Nowadays, despite a negative impact on the natural environment, coal combustion is still a significant energy source. One way to minimize the adverse side effects is sophisticated combustion technologies, such as, e.g., staged combustion, co-combustion with biomass, and oxy-combustion. Maintaining the combustion process at its optimal state, considering the emission of harmful substances, safe operation, and costs requires immediate information about the process. Flame image is a primary source of data which proper processing make keeping the combustion at desired conditions, possible. The paper presents a method combining flame image processing with a deep convolutional neural network (DCNN) that ensures high accuracy of identifying undesired combustion states. The method is based on the adaptive selection of the gamma correction coefficient (*G*) in the flame segmentation process. It uses the empirically determined relationship between the *G* coefficient and the average intensity of the R image component. The pre-trained VGG16 model for classification was used. It provided accuracy in detecting particular combustion states on the ranging from 82 to 98%. High accuracy and fast processing time make the proposed method possible to apply in the real systems.

## 1. Introduction

One of the negative side effects of rapid technological development is an increase in environmental pollution. This is mainly due to emission into the atmosphere of by-products of the energy production process, where hard coal is still the predominant raw material. To counteract this situation, alternative sources of energy are sought, including renewable fuels. One of the worth mentioning options is the co-combustion of pulverized coal and biomass. Such an approach enables using the existing combustion facilities after the proper adaptation and is economically advantageous. For both the chemical and physical properties of the compounds of the fuel mixture are different and unsteady in time, monitoring of the co-combustion process is highly recommended. Undesired states of the process, especially unstable combustion can result from improper parameters of the fuel mixture as well as malfunctions of the combustion facility. Therefore, recognition of different states of the combustion process plays an important role taking into account its effectiveness and safety.

It is important to obtain information on the combustion process with a minimum possible delay that classical approaches such as analysis of the flue gases cannot maintain. Using a flame image as a source of information practically eliminates the delay problem [1]. This method has other advantages. Firstly, it is non-invasive, and secondly—the information has controlled spatial resolution. The power boilers usually have several dozens of burners, which operation can be monitored independently when using image processing based methods.

Recently, the rapid advance of machine learning methods can be observed, especially deep neural networks. Various architectures of such networks have been developed, and many of them achieve excellent performance in image recognition tasks, including flame images. Examples include deep convolutional neural networks [2,3,4,5,6], deep belief networks [7,8], deep convolutional auto-encoder [9], deep convolutional auto-encoder connected with the principal component analysis and the hidden Markov model [10], deep, fully connected neural networks [11], deep convolutional selective autoencoder [12] and various architectures followed by a symbolic time series analysis [13,14]. Each of the architectures mentioned above plays a vital role in a specific application area. For example, deep convolutional neural networks are often used for image and speech recognition and for natural language processing. Deep belief networks reduce the data dimension. Deep convolutional auto-encoders reduce the data dimension, denoise the input data, impose a condition of rarity on the coding layer, randomly generate new data resembling the learning data. Deep, fully connected neural networks are used in different architectures’ final layers to perform the classification task. Although the use of deep learning methods is extensive, reports of their usage for diagnosing pulverized coal and biomass co-firing based on flame images cannot be described as widespread.

The problem we tried to solve in the research presented was that together with the fuel, a certain uncontrolled amount of air always gets into the combustion chamber, which changes the standard (effective) value of the excess air coefficient. As a result, the combustion conditions change, and the fuel ceases to be completely incinerated. Based on the information about a new undesirable state, it is possible to restore the normal (proper) conditions of the combustion process. Our research results allow us to obtain such information by analyzing and classifying the flame image using DCNN. It is a new approach in research on the diagnosis of pulverized coal and biomass co-firing. We are not aware of the research in which deep learning algorithms would be used to solve this problem. The proposed method significantly simplifies the stage of image data processing compared to traditional machine learning algorithms because stages such as data pre-processing, feature engineering, feature extraction and feature selection are eliminated. The DCNN can reveal the internal features of individual combustion states based on raw data obtained from images while ensuring high classification efficiency [15,16,17]. Additionally, the relatively low consumption time of the method used creates a chance for its use in industrial combustion systems.

Before applying DCNN, we had to segment the flame area and then extract the region of interest (ROI). It is not a trivial problem because of the high variability of both the shape and texture of the flame. In addition, segmentation is hindered by the background effect, the brightness of which varies depending on the current thermal power in the combustion chamber. To deal with this problem, a non-linear gamma correction has been applied. The brighter image pixels (belonging to the flame) were only slightly dimmed, whereas the darker pixels (those belonging to the background) were darkened to a much greater extent. Thus, the background impact on segmentation quality was weakened. The magnitude of change in the intensity of individual pixels depended on the value of the gamma correction coefficient (*G*). In the work, an adaptive selection of this value was applied, based on the intensity of the red component of the current image frame. The form of the function used to calculate the required value of *G* was found experimentally. It should be emphasized that the gamma correction was used only to obtain the most faithful contour of the flame, regardless of the brightness of the background. The obtained contour was then overlapped on the original image (without correction) and based on this image the ROI was extracted, the center of which was in the contour’s centroid. Detailed description of this method can be found in Section 2.3 and Section 2.4.

## 2. Materials and Methods

### 2.1. Experimental Setup

**Figure 1:**
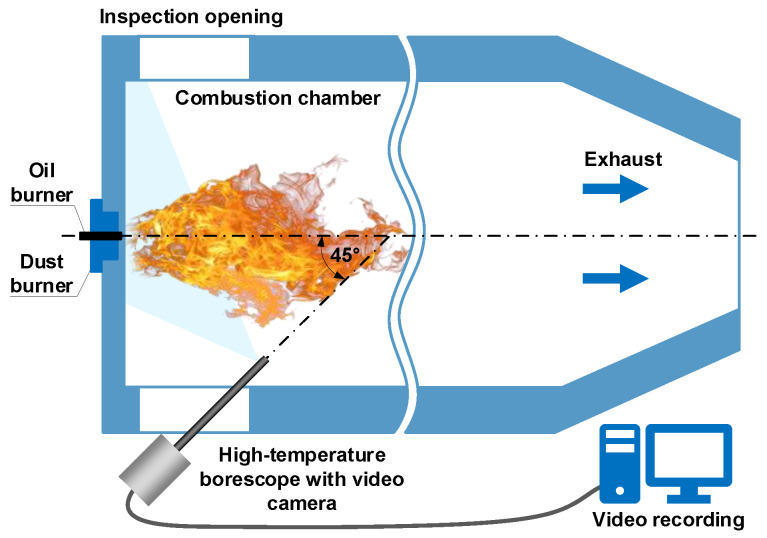
Overview of the camera mounting in the combustion chamber.

Investigations of pulverized coal and shredded biomass co-firing were carried out at a laboratory stand, built of a cylindrical combustion chamber with a length of approximately 2.5 m and a diameter of approximately 0.7 m. A digital camera (Mikrotron MC1311 by Mikrotron GmbH, Unterschleissheim, Germany) with a full-frame transfer matrix equipped with a CamerLink interface was used to record the flame image. Its main parameters are CMOS image sensor with a resolution of 1280 × 1024 pixels, a dynamic range of 59 dB, sensitivity of 1600 bit/lux⋅s at 550 nm, acquisition speed of 500 frames/s at full resolution. The video camera was attached to a high-temperature borescope installed in the chamber sight glass at an angle of about 45° to the burner’s axis (Figure 1). Image frames of 800 × 800 pixels were recorded, in RGB mode with 24-bit color depth. During recording, a fixed exposure time and a frame rate of 500 frames per second were maintained. Video recordings for individual combustion variants were saved in mass storage in lossless AVI files, with the duration of each recording being 300 s [18].

During the tests, co-firing of mixtures of pulverized coal and biomass in the form of crushed straw was carried out, with different air and fuel proportions. The measurements were made at a constant fuel flow so that the calculated thermal output of the burner (P_th_) was kept constant. The amount of incoming air was regulated, which affected the value of the excess air coefficient (*λ*). During the co-firing of the mixture of pulverized coal and biomass, the total combustion took place for *λ* ≈ 0.75; therefore this value of the excess air coefficient was considered the value corresponding to the "normal" combustion conditions. On this basis, three values of the *λ* coefficient were adopted in the studies. The first corresponded to normal conditions (*λ* = 0.75). The second value corresponded to the insufficient air (*λ* = 0.65), and the third to its excess (*λ* = 0.85). The tests were carried out for seven combustion variants, with different P_th_ and *λ* and where coal mixture with 10% of biomass (straw) has been examined (Table 1).

### 2.2. Statistical Analysis

**Figure 2:**
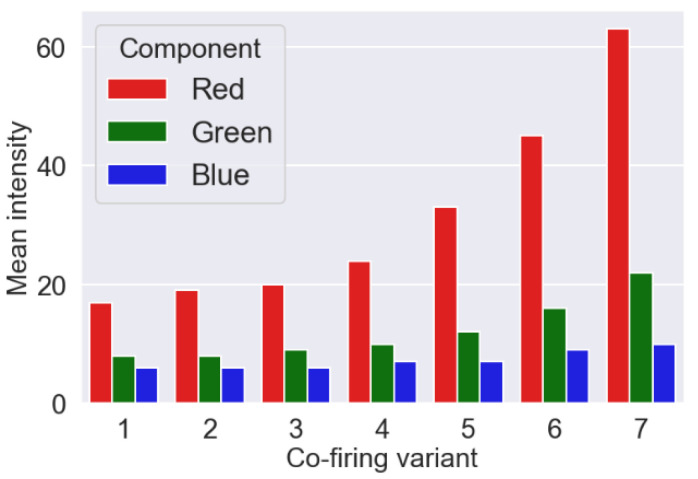
An average value of the color components for individual variants of the combustion process. The results presented in the graphs were obtained for the entire set of 1676 images for each variant.

During the measurements, the combustion process took place in stationary conditions regarding air and fuel flows and fuel mixture composition. On this basis, we considered that a fragment of the flame image recording with a duration of about 3 s was sufficient for use in further studies. Frames of video files, corresponding to various variants of the combustion process, have been saved in the form of images with a size of 800 × 800 pixels. Each video file has been converted to 1676 PNG image files with 24-bit color depth. Analysis of the contribution of individual color components in creating a flame image showed that the component of the red (R) color plays a fundamental role as shown in Figure 2.

**Figure 3:**
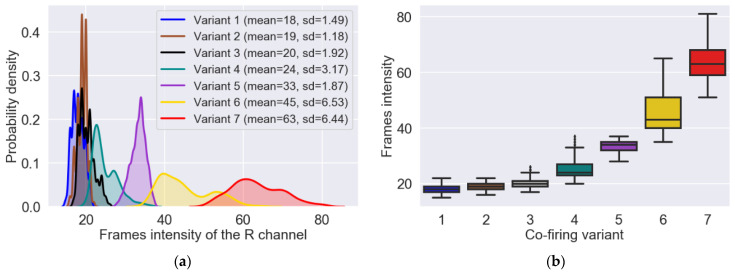
Probability density functions of the frames intensity (**a**) and box plots showing the dispersion of the frames intensity around the median (**b**). The results presented in the graphs were obtained based on the analysis of the entire set of 1676 images for each variant.

These charts were made for each combustion variant and show the average values of the individual components for the entire data set. Due to the dominant role of the R component, its intensity (i.e., the average value of all pixels) for each image was calculated. The probability density function for R component and each combustion variant and corresponding box plots were presented in Figure 3a,b, respectively.

### 2.3. Gamma Correction

At the beginning of the algorithm realizing the gamma correction, a lookup table (LUT) is built. During this operation, elements of the input array with values in the range [0, 255] are scaled to the range [0, 1.0]. The new values are then raised to power with an exponent equal to the inverse of the gamma correction coefficient. This operation is carried out according to the formula: *O* = *I*^1/*G*^, where: *I*—the value of the element of the input array; *O*—the value of the element of the output array; *G*—gamma correction coefficient. Finally, the obtained values are scaled back to the range [0, 255] and stored in the LUT table. The gamma correction is based on the fact that for each pixel of the input image, a mapping (new intensity value) is found in the LUT table. The set of all mappings creates the output image (after correction). Values of coefficient *G* > 1 brighten the image, no changes are made for *G* = 1, while for *G* < 1 the image is darker.

**Figure 4:**
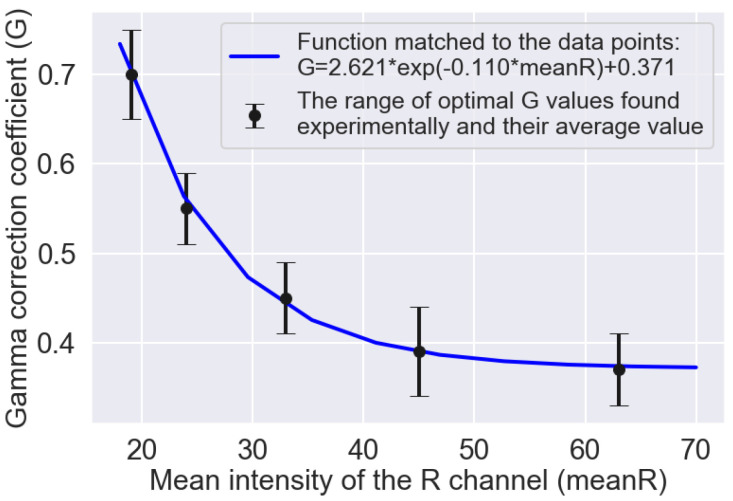
Optimal values of the gamma correction coefficient determined experimentally, based on the segmentation quality, for the mean intensities of the R channel of individual variants (black points). The blue line shows the course of the exponential function matched to the data points.

In the studies, an adaptive selection of the gamma correction coefficient value was applied, depending on the intensity of the R component of the current image frame. The statistical analysis allowed to determine mean values of frames intensity (meanR) for particular variants: Variant 1—18 ± 1.49, Variant 2—19 ± 1.18, Variant 3—20 ± 1.92, Variant 4—24 ± 3.17, Variant 5—33 ± 1.87, Variant 6—45 ± 6.53, and Variant 7—63 ± 6.44. On this basis, for each variant, ten images were found for which the intensity of the R component was equal to the average intensity determined earlier. Due to the similar mean intensity values, for Variants 1–3 one value was assumed equal to 19. Then, for each of the ten images selected for the given variant, flame segmentation was performed during which the optimal value of the gamma correction coefficient was determined arbitrarily. The averaging of determined experimentally ten results, allowed obtaining the optimal values of the *G* coefficient. This way, the coordinates of five points (mean*R*, *G*) were found to which the following exponential function was matched: *G* = 2.621 ∗ e^−0.110^ ^∗ mean*R*^ + 0.371 (Figure 4). This function was further used in the segmentation process to adaptively select the value of the gamma correction coefficient, depending on the intensity of the R component of the current image frame.

**Figure 5:**
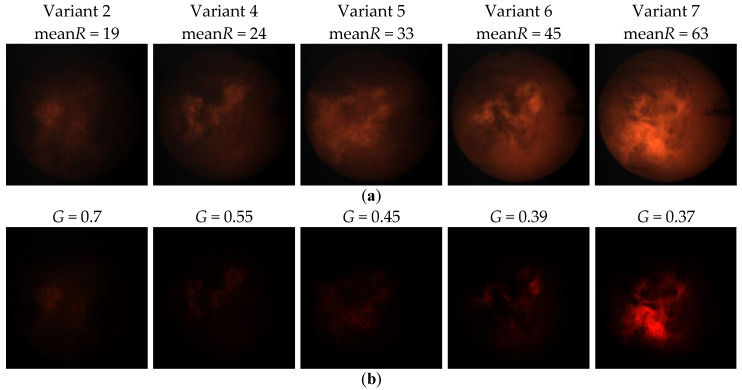
Gamma correction for exemplary flame images belonging to different variants: (**a**) input images; (**b**) images after gamma correction. The images show that the adaptive selection of the gamma correction coefficient effectively eliminates the image background, regardless of the average intensity of the R component. Thanks to this, better conditions for segmentation of the flame area are created.

**Figure 6:**
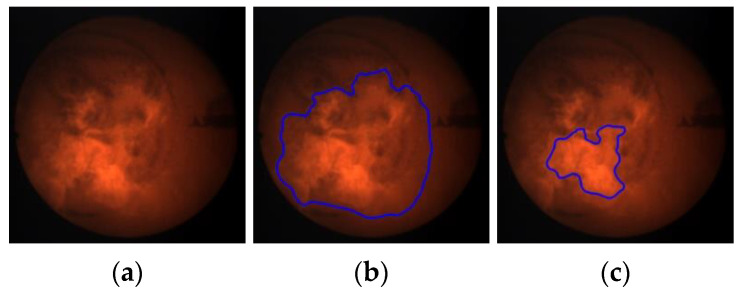
Impact of gamma correction on the segmentation quality: (**a**) exemplary flame image belonging to Variant 7; (**b**) segmentation result without gamma correction (the segmented object includes also the large background area); (**c**) segmentation result after gamma correction.

Figure 5 presents the results of gamma correction for sample flame images belonging to different variants. The upper row (Figure 5a) shows the input images that correspond to the different combustion variants and the different intensity values of the R component. Observing these images, you can see that there is a stronger and stronger background effect for the subsequent combustion variants (from left to right). Consequently, the area of the flame is more and more strongly masked by the hot walls of the combustion chamber. The bottom row (Figure 5b) shows the input images after correction according to the formula defined empirically (Figure 4). It can be seen that the adaptive selection of the gamma correction coefficient effectively eliminates the background of the image, regardless of the average intensity of the R component. It creates better conditions for segmentation of the flame area, as shown in Figure 6 where one can notice differences in the segmentation results of the same flame image (Figure 6a) without and after gamma correction. The result of the segmentation without correction contains significant background area (Figure 6b). While the use of adaptive gamma correction significantly improves the quality of the flame area segmentation by eliminating the background (Figure 6c).

### 2.4. Image Processing

All operations related to image processing were performed using Python 3.7.3 and libraries OpenCV 3.4.1 and NumPy 1.16.4. The purpose of the processing was to extract the square ROI, the center of which was the centroid of the flame contour. The square shape of the ROI resulted from the requirements set by the classifier used. Regarding the size of the ROI, two opposite necessities had to be taken into account. On the one hand, this size should be as large as possible, because then the ROI contains a lot of information that allows to effectively distinguish between different variants. However, on the other hand, the smaller the ROI, the shorter the processing time and the smaller frame losses. These losses occur when the extraction condition is not met, i.e., the ROI is not entirely contained in the flame contour. To determine the percentage of picture frames for which the extraction condition was not met, the segmentation algorithm for different sized ROIs was tested. The results obtained are presented in Table 2.

The problem of frame losses is very important because the flames belonging to various variants are different. In the case of Variants 1–3, the flame area is relatively large and regular, and the image shows a low dynamics in texture changes. These changes occur on a large surface, so to capture differences between variants, the ROI must be large enough. For the Variants 4–7, the flame area is more irregular with higher dynamics in texture changes. Therefore, to make frame losses smaller, the ROI size must be smaller than for Variants 1–3. In the case of Variants 4–7, texture changes between consecutive frames occur on a relatively small area, therefore a smaller ROI size contains enough information to ensure effective classification. During the tests, it was assumed that frame losses cannot exceed 15%. Taking into account this assumption and previous considerations, in further research the size of ROI equal to 100 × 100 pixels for Variants 1–3 and 40 × 40 pixels for Variants 4–7 was adopted.

The most important operations performed during image processing are listed below.

**Figure 7:**
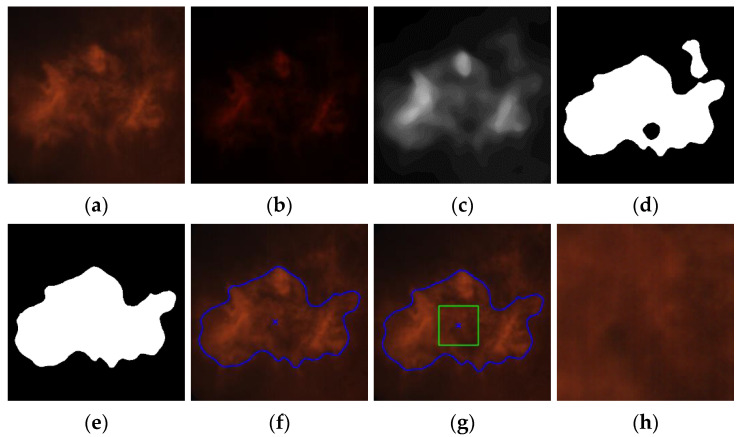
Results of the most important operations performed during image processing: (**a**) source image; (**b**) the result of the gamma correction according to the function *G* = f(meanR) (Figure 4); (**c**) greyscale image after histogram normalization and median filtering; (**d**) binary image after thresholding with the Otsu method; (**e**) image after morphological operations (closing, opening) and finding the contour with the largest area; (**f**) the image with the contour of the flame marked and its centroid (blue cross); (**g**) image with the ROI marked (green square of a size of 100 × 100 or 40 × 40 pixels), the center of which is in the contour’s centroid; (**h**) the ROI copied from the source image (without gamma correction). For greater readability, the above images show the central part of the images processed that include the flame. The sizes of all images (except the ROI) were 800 × 800 pixels.

1. Reading a source image in PNG format, with 24-bit color depth and size of 800 × 800 pixels (Figure 7a).
2. Calculating the intensity of the R component of the image as the average value of all its pixels.
3. Performing gamma correction according to the procedure described in Section 2.3 (Figure 7b).
4. Image conversion to 8-bit greyscale.
5. Image histogram normalization.
6. Median filtration with a mask size of 33 × 33 pixels (Figure 7c).
7. Image thresholding using the Otsu method (Figure 7d). The results of several segmentation methods were compared, including Otsu thresholding [19], Bradley thresholding [20], level set method, adaptive thresholding, and active contour method.
8. Morphological operations (closing, opening) with the size of a structural element equal to 3 × 3 pixels and detecting all contours in the image.
9. Finding the contour with the largest area (Figure 7e). We took advantage of the observation that statistically, among all contours present in the image, the one belonging to the flame has the largest surface area.
10. Calculation of the contour’s centroid coordinates (Figure 7f). The coordinates of the contour’s centroid were calculated using the formulas: *x* = m_10_/m_00_, *y* = m_01_/m_00_, where: m_00_—zero order normal moment, m_10_, m_01_—the first order normal moments.
11. Finding a square of a size 100 × 100 or 40 × 40 pixels (depending on the image variant) the center of which is in the contour’s centroid (Figure 7g).
12. Copying the ROI determined by the found square from the source image (without gamma correction) provided that it is completely contained in the flame contour (Figure 7h). If the condition is not met, the ROI extraction is not carried out.

### 2.5. Deep Convolutional Neural Network

The Keras library includes many models for image classification (Xception, VGG16, VGG19, ResNet, ResNetV2, ResNeXt, InceptionV3, InceptionResNetV2, MobileNet, MobileNetV2, DenseNet, NASNet). All these models have been trained on the ImageNet collection containing approximately 1.4 million images divided into 1000 classes [21]. In our studies, the number of images belonging to individual variants was 1676. In the context of deep neural network learning, this is a small data set. In such cases, the practice of using a network that has been previously trained on a large data set is often used [22]. If the set was large enough and general enough, the spatial hierarchy of features learned by the previously trained module could effectively play the role of the general model for image processing. The features of this network can be helpful when solving various problems of image processing, despite the fact that new problems require the recognition of completely different classes than was the case with the original task.

**Figure 8:**
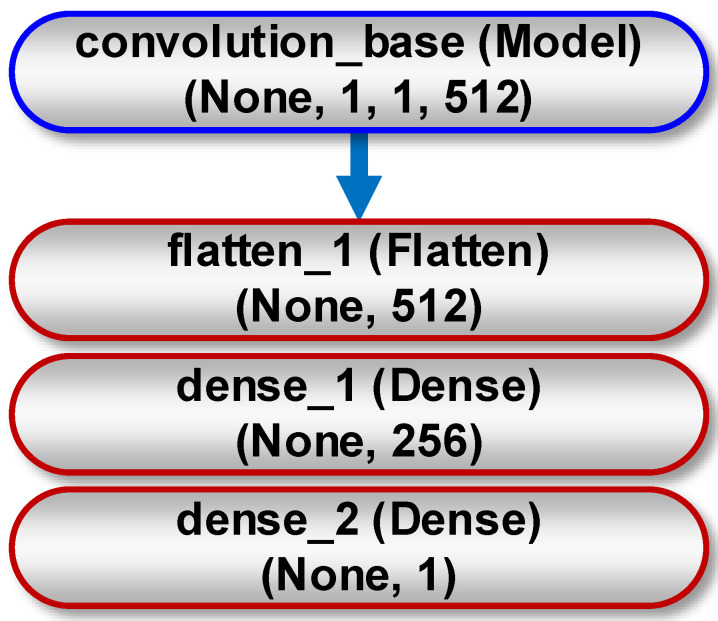
General scheme for implementing deep neural network consisting of a convolutional base (blue line) and own classifier (red line) for binary classification. The meaning of the Keras classes visible on the individual layers is as follows: Flatten—converts a 3-dimensional tensor to a 1-dimensional vector; Dense—densely connected layer. The feature map has a shape (samples, height, width, channels). The None parameter corresponds to any number of samples.

The research was based on the VGG16 architecture developed by Karen Simonyan and Andrew Zisserman in 2014 [23]. This model was chosen because of its simplicity (it has the lowest topological depth among the models available in the Keras library). It consists of two parts—the first one is a convolutional base, built from a series of connecting layers (MaxPooling2D) and convolutional layers (Conv2D). The second part is a densely connected classifier located at the end of the network. The study used a convolutional base of the VGG16 model, because representations it taught present general concepts that are suitable for solving various problems related to image processing. Instead of the original classifier, we used our own classifier, built of two "Dense" layers (Figure 8). This is because of the fact that the representations it learned are specific to the tested set of classes (variants of the co-firing process) on which the model was trained.

**Figure 9:**
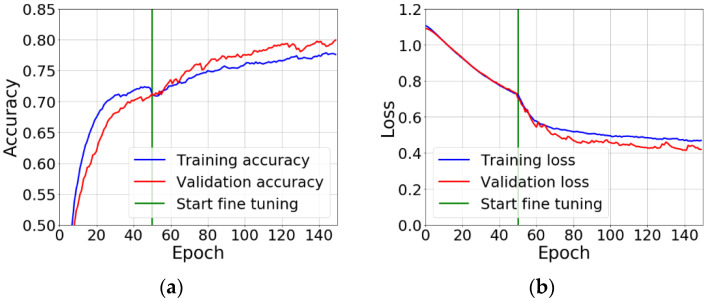
Sample plots of the accuracy (**a**) and loss (**b**) of training and validation processes for Variant 1 vs. 2 vs. 3. During the first 50 epochs, new layers of the network are trained, while the whole convolutional base is frozen (feature extraction). Then the fine tuning starts, during which the upper layers of the convolutional base are unfrozen and tuned for a period of 100 epochs together with the new classifier.

The network training was carried out in two phases (Figure 9). The first one was feature extraction—the convolutional base has been frozen, and the added layers creating a new classifier, were initiated randomly and trained over a period of 50 epochs using data augmentation. The second one was fine tuning—the upper layers of the convolutional base (layers 16–19) have been unfrozen and trained for a period of 100 epochs together with new layers, also using data augmentation. At the end, the entire convolutional base has been unfrozen.

Augmentation consisted of performing random, vertical, and horizontal transformations of the image, rotation, cropping, zooming in and reflecting half of the image in a horizontal plane. As a loss function, binary cross entropy for binary classification and categorical cross entropy for multi-class classification were applied. The optimization algorithm was root mean square propagation (RMSProp) with a low value of the learning parameter. It was 2 × 10^−5^ for the feature extraction phase and 1 × 10^−5^ for the tuning phase. The low value of this parameter resulted from the fact that modifications of the representation of the three tuned layers of the convolutional base had to be minimized. Too large changes in these values could harm data representations. The classification accuracy was applied to evaluate learning results. In the last layer of the classifier, "sigmoid" for binary classification and "softmax" for multi-class classification were used as activation functions.

The specification of the hardware and software environment used to build the models was as follows:
CPUIntel Core i5-3470 3.20 GHz32 GB RAMGPUNVIDIA GeForce GTX 10603 GB RAMOperating systemWindows 1064-bitPlatformTensorFlow [24]1.13.1PlatformCUDA9.0LibraryKeras [25]2.2.4LibrarycuDNN7.0Programming languagePython3.7.3

The construction time of the multi-class classification model, detecting Variants 1–3 (image size of 100 × 100 pixels), was about 34 min. In the case of the binary classification model, for Variants 1 and 2 (image size of 100 × 100 pixels), this time was about 23 min. In turn, for models recognizing Variants 4 and 5 as well as 6 and 7 (image size of 40 × 40 pixels), the model construction time was 7 min. Table 3 shows the number of cases belonging to individual variants used for models building.

## 3. Results

**Figure 10:**
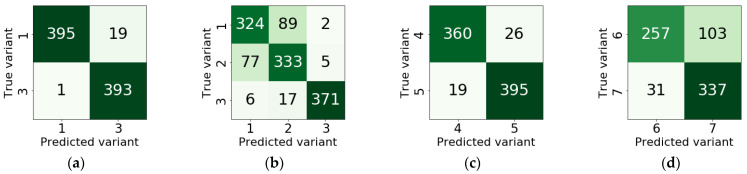
Confusion matrices: (**a**) Variant 1 vs. 3; (**b**) Variant 1 vs. 2 vs. 3; (**c**) Variant 4 vs. 5; (**d**) Variant 6 vs. 7.

Four classifier models were built that implemented the following classification options: Variant 1 vs. 3, Variant 1 vs. 2 vs. 3, Variant 4 vs. 5 and Variant 6 vs. 7. Images of a size of 100 × 100 pixels were used in the first two models, while the last two models utilized images of a size of 40 × 40 pixels. In each case, the entire set of images was randomly divided into training, validation and test subsets (Table 3). As a result of models testing, classification accuracy (ACC), loss, F1-score, recall and precision were calculated (Table 4). Additionally, confusion matrices have been determined that provide information on the errors made by the model when assigning observations to individual combustion variants (Figure 10).

All constructed classifier models are characterized by relatively high accuracy. In the case of binary classification, the most accurate was the model that distinguished variants 1 and 3 (ACC = 0.98). The classifier of Variants 4 and 5 took second place (ACC = 0.94). However, the smallest accuracy was obtained for the model built for Variants 6 and 7 (ACC = 0.82). This regularity is also reflected by the remaining measures of the classification quality, i.e., precision, recall, F1-score and loss. More detailed information on the recognition accuracy of individual variants is given in confusion matrices (Figure 10). We can see that the worst situation is for Variant 6, in which case 29% of observations were classified as Variant 7. For the remaining variants, the accuracy of the correct classification was greater than 92%.

**Figure 11:**
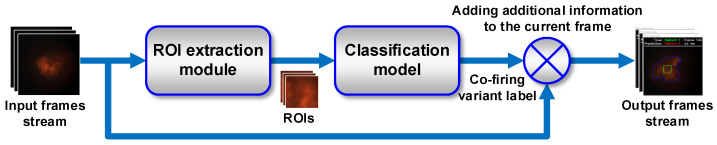
The general principle of operation of the application for testing the method of identifying variants of the co-firing process.

To test our flame segmentation method, we developed an application for identifying variants of the co-firing process. The application was implemented in Python 3.7.3, using OpenCV 3.4.1, NumPy 1.16.4, Keras 2.2.4. and TensorFlow 1.13.1. The first goal of the application construction was to create a prototype that could work on the computer controlling the flame monitoring camera in the combustion chamber. The second goal was to determine the average number of frames processed per second for typical PCs that could work as a workstation. The diagram presenting the general principle of application operation is shown in Figure 11.

**Figure 12:**
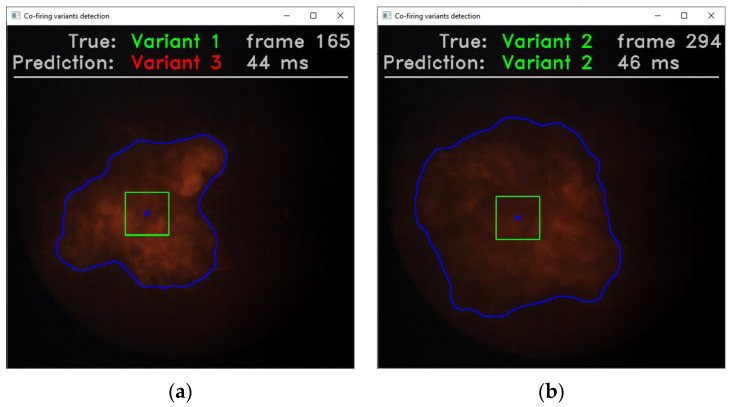
Application interface for testing the method of identifying co-firing variants: (**a**) an example of incorrect classification; (**b**) an example of correct classification. At the top, the following information is displayed: actual label of the currently displayed variant (green color), below—a label of the variant recognized by the classifier (green color—correct classification, red—incorrect), to the right—current image frame number, below—the frame processing time, including the time of the ROI extraction and the co-firing variant prediction time using the constructed model. In the central part, the contour of a flame and its centroid (blue cross) are marked as well as the ROI (green square), the center of which is in the contour’s centroid.

During the application testing, different variants of the co-firing process were simulated by sequential loading of the corresponding video files saved on the hard disk (Figure 12). Each sequence contained 200 initial frames of the original video recording, so the total number of frames used in the test was 600 for Variants 1–3 and 400 for Variants 4 and 5. Displaying the predicted label of the co-firing variant was the main objective of the application, but additional information was also presented, helpful in assessing the operation of the classifier model as well as the application itself. This information included: the real label of the co-firing variant, the number of the current image frame and the processing time.

Table 5 summarizes the test results in which the average times of: the ROI extraction (*t*_ext_), co-firing variant prediction (*t*_pred_) and overall processing (*t*_proc_) were measured for two computer systems on which only the necessary system processes were running. The systems used had the following specifications:

- PC1—Intel Core i5-3470 3.20 GHz, 32 GB RAM, NVIDIA GeForce GTX 1060 3 GB, Windows 10 64-bit;
- PC2—Intel Xeon E5-2660 2.60 GHz, 32 GB RAM, NVIDIA Tesla K20c 5 GB, Ubuntu 18.04.2 LTS 64-bit.

## 4. Discussion

The test results showed that the ROI extraction time is about three times higher than the co-firing variant prediction time. Additional frame processing time (not included in the test) is negligible in relation to the *t*_ext_ and is about 3 ms. Thus, in an effort to reduce the overall frame processing time *t*_proc_, one should try to optimize the ROI extraction algorithm. Another possibility is to run this algorithm on the GPU, then the OpenCV library (used for image processing) should be able to cooperate with the CUDA platform. On the basis of the conducted test, it can be concluded that the used, exemplary computer systems, process about 20 frames per second, and the size of the processed images is not significant. In turn, the entire pulverized –coal and biomass co-firing system is characterized by a certain time constant. It can be understood as the time elapsing from the moment of changing the system operation parameters, e.g., the excess air coefficient *λ*, until the moment when the process in the combustion chamber stabilizes. Based on the observations of the real industrial systems, we can say that this time ranges from several seconds to several minutes, so it is larger than the frame processing time from two to three orders of magnitude. Thus, the frame processing time on the level of 44–52 ms seems sufficient to allow the presented flame segmentation method to be practically applied. At the same time, it can be concluded that computer systems with parameters similar to those used in the test can be used at the workstation to monitor the combustion process.

It should be noted that in the literature we did not find results concerning the solution of the same classification problem as the presented. Therefore, as part of the comparison, we refer to the results obtained when solving relatively similar (but not identical) problems (Table 6). Comparing our own results with those of other authors, we can state that they are at a similar level to those of other authors.

## 5. Conclusions

A flame contour segmentation method was developed, based on the adaptive selection of the gamma correction coefficient (*G*), depending on the current thermal power. The method used the empirically determined relationship between the *G* coefficient and the average intensity of the R component. Based on the VGG16 architecture, four classifier models were built. Their average accuracy, for individual combustion variants, was as follows: 1 vs. 3—98%, 1 vs. 2 vs. 3—84%, 4 vs. 5—94%, and 6 vs. 7—82%. A prototype application for recognizing co-firing variants online let us evaluate the time-consuming nature of the solutions applied. For the two test computer systems, the average frame processing time ranged from 44 to 52 ms. Processing of approximately 20 frames per second seems sufficient in a context of the delays occurring in the real combustion systems. Based on the information about the appearance of a new undesirable state, normal conditions of the combustion process could be restored.

## Figures and Tables

**Table 1:** Variants of the combustion process. P_th_—thermal power, *λ*—excess air coefficient.

Variant	P_th_ (kW)	*λ*	Fuel Flow (kg/h)	Secondary Air Flow (Nm^3^/h)
1	250	0.85	35.2	103.6
2	250	0.75	36.0	73.2
3	250	0.65	39.4	62.9
4	300	0.85	44.2	132.5
5	300	0.75	43.5	96.4
6	400	0.75	59.7	181.3
7	400	0.65	56.8	152.8

**Table 2:** Percentage of image frames discarded due to failure to meet the region of interest (ROI) extraction condition depending on the ROI size. This condition means that the ROI must be completely contained in the flame contour.

Variant	100 × 100	80 × 80	60 × 60	40 × 40
1	0.12	0.0	0.0	0.0
2	0.06	0.0	0.0	0.0
3	6.03	2.27	0.89	0.36
4	36.58	23.75	14.86	7.88
5	2.45	1.37	0.78	0.12
6	44.81	30.43	21.54	14.14
7	39.74	27.15	18.08	11.75

**Table 3:** Number of cases belonging to particular variants.

Dataset	1	2	3	4	5	6	7
Full	1670	1671	1575	1544	1669	1439	1477
Training	837	837	787	772	836	719	739
Validation	418	419	394	386	419	360	370
Testing	415	415	394	386	414	360	368

**Table 4:** Classification results.

Model	Variant	Precision	Recall	F1-Score	Accuracy	Loss	Image size
Binary	1	1.00	0.95	0.98	0.98	0.06	100 × 100
	3	0.95	1.00	0.98			
Multi-class	1	0.80	0.78	0.79	0.84	0.40	
	2	0.76	0.80	0.78			
	3	0.98	0.94	0.96			
Binary	4	0.95	0.93	0.94	0.94	0.14	40 × 40
	5	0.94	0.95	0.95			
Binary	6	0.89	0.71	0.79	0.82	0.37	
	7	0.77	0.92	0.83			

**Table 5:** Application test results for identifying variants of the co-firing process. The test used the classifier model that detected Variants 1–3 and the model for Variants 4 and 5.

System	Variant	Image Size	*t*_ext_	*t*_pred_	*t*_proc_
PC1	1-2-3	100 × 100	31	10	44
PC2	1-2-3	100 × 100	35	14	52
PC1	4-5	40 × 40	32	9	44
PC2	4-5	40 × 40	36	11	49

The meaning of the symbols used in Table 5 is as follows: *t*_ext_—average ROI extraction time; *t*_pred_—average time of the co-firing variant prediction; *t*_proc_—average overall frame processing time. Average values were calculated as a result of processing a video sequence consisting of 600 frames (100 × 100 pixels) for multiclass model and 400 frames (40 × 40 pixels) for binary model. All the measured times are expressed in milliseconds.

**Table 6:** Comparison of the classification results, where the subjects of study were flame images.

Application	Architecture	ACC	Prec., Rec.	No. in Ref.
Recognizing variants of pulverized coal and biomass co-firing	DCNN	0.82–0.98	0.83–0.98, 0.81–0.98	Own results
Monitoring combustion quality (complete, partial, incomplete) in a coal-fired boiler	FLD+RBN	–	0.96, 1.0	[6]
Recognizing the conditions of the pulverized coal combustion	PCA+RWN	0.91	–	[12]
Identifying burning states of oil, powder and normal one	DCNN	1.0	–	[26]
Detecting instability in an experimental combustion system based on flame images and sound pressure	DCNN	0.69–1.0	–	[3]
Abnormal condition detection in the experimental combustion system	DBN	0.96	–	[8]
Determination of combustion regimes using flame images of a gas burner	DCNN	0.98	–	[2]
Detection of the stable, semi-stable and unstable combustion status in a thermal power plant	DCAE+PCA+HMM	0.97	–	[10]

DCNN—Deep convolutional neural network; DBN—Deep belief network; DCAE+PCA+HMM—Deep convolutional auto-encoder, principal component analysis and hidden Markov model; PCA+RWN—Principal component analysis and random weight network; FLD+RBN—Fisher’s linear discriminant analysis and radial basis network; Prec.—precision; Rec.—recall.

## Data Availability

No new data were created or analyzed in this study. Data sharing is not applicable to this article.
